# The experiences of caring for someone with dementia and a learning disability: A qualitative systematic review

**DOI:** 10.1177/14713012231225797

**Published:** 2024-01-03

**Authors:** Michelle Hughes, Kerry Hanna, Akpevwoghene Wiles, Ellie Taylor, Clarissa Giebel

**Affiliations:** Department of Primary Care and Mental Health, 4591 University of Liverpool, UK; School of Health Sciences, 4591 University of Liverpool, UK; Department of Primary Care and Mental Health, 4591 University of Liverpool, UK; 4586 Mersey Care NHS Trust, UK; Department of Primary Care and Mental Health, 4591 University of Liverpool, UK; NIHR Applied Research Collaboration North West Coast, UK

**Keywords:** dementia, learning disabilities, workforce, caregiver

## Abstract

**Background:**

The life expectancy of people with a learning disability is increasing and with this comes a greater risk of developing dementia. Dementia poses new challenges for both family and formal learning disability carers as they try to support dementia’s progressive nature and quality of life for their care recipient. This qualitative systematic review explores the evidence base of family and formal carers’ experiences and needs of caring for someone with both a learning disability and dementia.

**Methods:**

Six electronic databases (PubMed, PsycINFO, Cochrane Library, Prospero, Scopus, CINAHL), were searched in May 2022, utilising a predefined search strategy. Thirteen papers fulfilled inclusion criteria and were included in in the review.

**Results:**

Thematic synthesis was used to explore and synthesise the qualitative findings of the studies. Four conceptual themes were identified following analysis: *Knowledge and skills, Accessing support, Repercussions of dementia for carers, Influences of continuity of caring role*.

**Conclusion:**

There are significant training and educational needs for all carers who support the dual diagnosis of dementia and learning disability. Differences between family and formal carers relate to the organisational support and process available to formal carers. Parity across services combined with sufficiently trained carers may support dementia diagnosis and improve quality of care provided. Further research is needed to address environmental, and economic barriers carers face to facilitate ageing in place for their care recipients.

## Introduction

Dementia is an umbrella term for multiple different subtypes, including Alzheimer’s disease dementia, vascular dementia, and Lewy Body dementia, to name a few. There are over 100 subtypes of dementia (Alzheimer’s Disease International, 2020a), which can significantly affect areas of functioning such as language, orientation, cognition and emotional control (Alzheimer’s Disease International, 2020b; Smits et al., 2015), as well as impact on behaviour, physical functioning, and the ability to perform everyday activities (Giebel et al., 2014). There are common symptom similarities between dementia subtypes, although dementia will impact every individual differently (Alzheimer’s Disease International, 2020b). In the UK, there are an estimated 920,000 people living with dementia in the UK and this figure is set to rise to over a million by 2025 (Wittenberg et al., 2019). This estimated figure will include individuals who also have a learning disability.

A learning disability is a lifelong condition, often characterised as reduced intellectual ability and social functioning that impacts a person’s ability to undertake daily tasks and live independently (Department of Health, 2001). Direct care to cultivate independence and support care needs is often provided by family carers, such as parents and siblings within their home environment, and formal carers, who are employed to deliver care within a range of residential settings (Herron et al., 2020). Individuals with a learning disability have historically been marginalised and subject to discrimination and human right violations (Joint Committee on Human Rights, 2008). Significant health inequalities are found within the learning disability population with individuals having poorer physical and mental health compared to their peers (White et al., 2022). Such disparities can be attributed to insufficient access to and delivery of healthcare provision and social, economic and environmental inequities (Emerson et al., 2011; Nocon et al., 2008; Wilson et al., 2017).

Due to the increased life expectancy of people with a learning disability, there is an increased risk of developing dementia, especially those who have Down’s syndrome (DS) (Dementia UK, 2021; Heller et al., 2018; McCarron et al., 2017; Strydom et al., 2010; Takenoshita et al., 2020). Dementia prevalence rates are higher for individuals with a learning disability compared to the general population (Bayen et al., 2018; Burke et al., 2018; Strydom et al., 2013). Furthermore, there are several important differences to consider for people who also have a learning disability such as earlier onset of dementia, quicker progression, comorbid health conditions, delayed diagnosis due to pre-existing impairment and differing presentation (Alzheimer’s Society, 2019).

Research highlights that carers for people with dementia alone already have very high unmet needs (e.g., education regarding dementia, mental health and external and practical support (Black et al., 2013; Gaugler et al., 2004; Jennings et al., 2015; Li, 2012; Philp et al., 1995). Developing dementia in addition to a learning disability has a compounding impact on the individual, their family and various other support systems (Llewellyn, 2011). Implications include the impact on quality of life, psychological and physical health, and financial and relationship stressors (Marsack-Topolewsk & Samuel, 2020; McLaughlin & Jones, 2011). The education and training that dual diagnosis carers receive to recognise the development and support the functional changes and progression of the dementia, through to end of life is reportedly lacking (Courtenay et al., 2010; Herron et al., 2020; McCarron et al., 2010). Such findings highlight the need to explore the current evidence base to generate a better understanding of the needs and experiences of family and formal carers of people with a dual diagnosis of dementia.

Existing systematic reviews pertaining to the dual diagnosis and caring have focused on professional carers (inclusive of healthcare professionals) experiences (Cleary & Doody, 2017) and care provision and interventions to meet the needs of people with a dual diagnosis and their carers (Courtenay et al., 2010). These reviews fail to fully capture the experiences of the primary caregivers for people with a learning disability and dementia. A recent small-scale review has been published (Acton et al., 2023) claiming to explore carers experience of caring for individuals with a learning disability and dementia. However upon review it would appear that Acton et al. (2023) have not explicitly followed their eligibility criteria for their qualitative synthesis, (e.g., quantitative and non-dementia papers included) and therefor findings should be interpreted with caution. The aim of this qualitative systematic review was to explore the evidence base of family and formal carers’ experiences of caring for someone with both a learning disability and dementia and their needs, and addressed the following research question: How do family and formal carers experience caring for someone with both a learning disability and dementia? Generating a deeper understanding of these caregivers’ experiences may enable professionals and services to tailor support for them and subsequently promote the care and the quality of life of those with a dual diagnosis.

## Method

The review question and search terms were developed utilising the PICO model (see Table 1) to ensure a fully comprehensive search (Methley et al., 2014). The review was registered on PROSPERO prospectively (CRD42022323477).

**Table 1.** PICO framework.

PICO Components	Criteria	Search Syntax
**P**opulation	Carers	caring OR carer OR “support worker” OR caretaker* OR caregiver* OR “care provider*” OR guardian* OR parent* OR famil* OR relative* OR care-giver* or spous* or husband* OR wife* OR wive* OR partner* OR mother* OR father*
**I**ntervention or exposure	Caring for people who have a dual diagnosis of a Learning disability and dementia	
**C**omparison	Qualitative	AND“mentally handicap*” OR “intellectual* impair*” OR “mental* retard*” OR “learning disabilit*” OR “developmental disabilit*” OR “developmental disorder*” OR “intellectual disabilit*” OR “intellectual developmental disorder*” OR “down* syndrome” OR “Trisomy 21”
**O**utcome	Experiences	AND dement* OR alzheimer*

### Search strategy

Six electronic databases (PubMed, PsycINFO, Cochrane Library, Prospero, Scopus, CINAHL), were searched in May 2022 and updated in July 2023 by one researcher (MH). A predefined search strategy was used which incorporated Boolean operators and truncation to optimise article retrieval. In addition to PICO the search terms were identified through exploration of existing research papers focused in this area and librarian consultation.

### Inclusion/Exclusion criteria

Journal articles describing carer experiences of caring for adults who have a learning disability (inclusive of Down’s Syndrome) and dementia where searched. English literature articles available from January 2000 to July 2023 were extracted. This timeline was chosen as it captured key time periods for learning disability recognition and rights awareness, government legislation and policy development (Department of Health, 2001, 2009; Flynn, 2012; Joint Committee on Human Rights, 2008; Murray, 2007).

Table 2 presents the applied inclusion and exclusion criteria applied for journal selection. Only qualitative data reported in peer-reviewed journal articles was included. The study population inclusion criteria encompassed carers aged 18 years and above, who were family carers (e.g., parent, sibling, cousin), or formal carers (any paid carers/within adult social care-supported living, day services and residential settings/non healthcare professionals) who provided care for a person with dual diagnosis of a learning disability and dementia. Studies that included both carer and health professional experiences were included if carer experiences could be separately identified and extracted.

**Table 2.** Journal article selection criteria.

Inclusion criteria	Exclusion Criteria
Qualitative research, peer-reviewed journal articles, qualitative data extracted from mixed method.	Quantitative research, non-peer reviewed articles.
Carers ≥18 years old	Carers <18 years old,
Family carer (e.g., friend, parent, sibling, cousin),	Studies without distinguishable family or formal carer experiences
Formal carers (any paid carers/within adult social care/non healthcare professionals).	Healthcare Professional’s experiences.
Studies exploring or highlighting carer experiences, perspectives of caring for a care recipient with a dual diagnosis of a learning disability and dementia	Studies exploring or highlighting carer experiences, perspectives, of caring for a care recipient without a dual diagnosis of a learning disability and dementia.

### Data extraction

**Figure 1.**
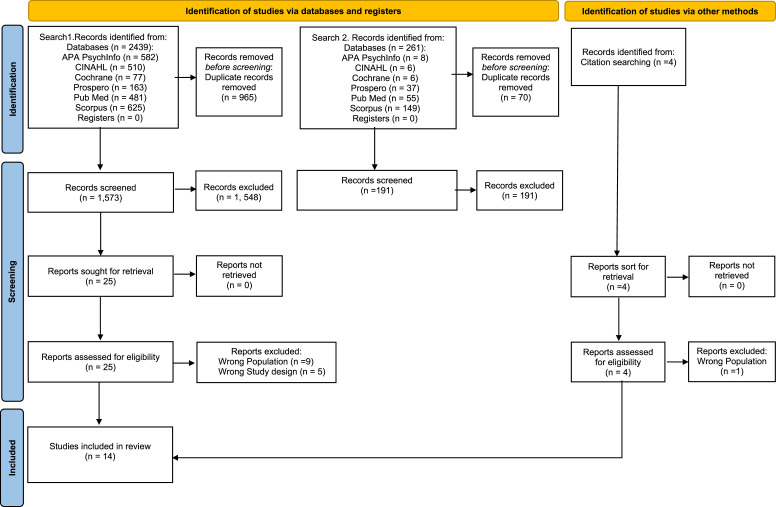
Prisma diagram depicting the screening process.

Database searches were combined and yielded 2438 records (see PRISMA Flowchart depicted in Figure 1). The web application Rayyan (Ouzzani et al., 2016) was used to remove duplicates (*n* = 865) and coordinate screening. At first stage of screening the title and abstracts of retrieved records (*n* = 1573) were assessed by one researcher (MH) using the inclusion and exclusion criteria above 10% of the included articles were cross checked by a member of the research team (AW) for agreement. Any disagreements were resolved in discussion with the wider research team (CG, KH). Articles which could not be included or excluded using abstract or title alone were subject to full text screening. Citations searches of 29 articles (25 retrieved database articles and 4 pertinent systematic reviews) identified four further potential records which were retrieved and reviewed for eligibility. At stage two, full text articles (*n* = 29) were screened independently by one researcher (MH), with a further 10% check conducted by another research team member (AW). Any uncertainties were discussed between the research team and resolved. Fourteen papers fulfilled inclusion criteria and were included in the review.

### Quality assessment

All included papers were quality assessed independently by two members of the research team (MH, ET). Any scoring discrepancies were discussed and resolved to ensure reliability. Included articles were assessed using the Standard Quality Assessment Criteria for Evaluating Primary Research Papers from a Variety of Fields (Kmet et al., 2004). Quality assessment was guided by 10 criteria checklist questions. For each question a study is awarded a score ranging from 0–2 dependent on how it’s considered to fulfil the criteria, (Yes = 2, Partial = 1, No = 0). The mean for each paper is then calculated to provide an overall summary score. A score of .75 and above indicated a good quality paper. An inclusion/exclusion cut-point was not applied, due to the limited research available within this area.

One paper used mixed methodology and so the Mixed Methods Appraisal Tool (MMAT) (Hong et al., 2018) was used to critically appraise it. This tool provides screening questions (*n* = 2) and a set of criteria (*n* = 5) for each methodology (quantitative, qualitative, and mixed method) by which scorers can assign either “yes”, “no” or “can’t tell”.

Tables 3 and 4 illustrate the quality assessment ratings for the qualitative studies. Low quality studies were not excluded but utilised in shaping the discussion of findings and conclusions (Sandelowski & Barroso, 2003). The recognised drawbacks of including such papers have been discussed under the limitations of the review.

**Table 3.** Quality assessment rating for identified qualitative papers (Kmet et al., 2004).

Authors	Q1.	Q2.	Q3.	Q4.	Q5.	Q6.	Q7.	Q8.	Q9.	Q10.	Summary Score
Carling-Jenkins et al. (2012)	2	2	2	2	2	2	1	1	2	0	.80
Coyle et al. (2014)	2	2	2	2	2	2	2	2	2	0	.90
Furniss et al. (2012)	2	2	2	2	2	2	2	0	2	0	.80
Herron et al. (2020)	2	2	2	2	2	2	2	2	2	1	.95
Iacono et al. (2014)	2	2	2	2	2	2	2	2	2	0	.90
Kerr and Wilkinson (2006)	1	2	2	2	2	1	2	2	2	0	.80
Marsack-Topolewski and Brady (2020)	2	2	2	2	2	2	2	2	2	0	.90
McLaughlin and Jones (2011)	2	2	2	2	1	1	2	2	2	0	.80
Perera and Standen (2014)	2	2	2	2	2	2	2	2	2	0	.90
Ryan et al. (2018)	2	2	2	2	2	2	2	2	2	0	.90
Sheth (2019)	2	2	2	2	2	2	2	0	2	0	.80
Watchman (2005)	2	2	2	2	1	1	0	0	2	0	.60
Wilkinson et al. (2005)	1	1	2	2	0	1	0	0	2	0	.45

**Table 4.** Quality assessment rating for identified mixed method paper (Hong et al., 2018).

Category of study designs	Methodological quality criteria	Yes	No	Can’t tell	Comments
Screening questions (for all types)	S1. Are there clear research questions?	Yes			
	S2. Do the collected data allow to address the research questions?	Yes			
1. Qualitative	1.1. Is the qualitative approach appropriate to answer the research question?	Yes			
	1.2. Are the qualitative data collection methods adequate to address the research question?	Yes			
	1.3. Are the findings adequately derived from the data?			Can’t tell	Small write up some themes not exemplified
	1.4. Is the interpretation of results sufficiently substantiated by data?	Yes			
	1.5. Is there coherence between qualitative data sources, collection, analysis and interpretation?	Yes			
4. Quantitative descriptive	4.1. Is the sampling strategy relevant to address the research question?	Yes			
	4.2. Is the sample representative of the target population?	Yes			
	4.3. Are the measurements appropriate?	Yes			
	4.4. Is the risk of nonresponse bias low?		No		Whole host of people not been asked. Convenience sample, web-based pilot study, which limited participants to those who had access to a computer and were familiar with the Internet.
	4.5. Is the statistical analysis appropriate to answer the research question?	Yes			
5. Mixed methods	5.1. Is there an adequate rationale for using a mixed methods design to address the research question?	Yes			
	5.2. Are the different components of the study effectively integrated to answer the research question?	Yes			
	5.3. Are the outputs of the integration of qualitative and quantitative components adequately interpreted?	Yes			
	5.4. Are divergences and inconsistencies between quantitative and qualitative results adequately addressed?	Yes			
	5.5. Do the different components of the study adhere to the quality criteria of each tradition of the methods involved?	Yes			

### Data synthesis

The extracted data were synthesised by one member of the research team (MH) and focused on country, population, sample size, and qualitative key findings only. Thematic synthesis (Thomas & Harden, 2008) was utilised to explore and synthesise the qualitative findings of the studies. This entailed an iterative 3-stage process of:

Line-by-line coding of the results section of included articles to generate initial codes; Rigorous grouping and regrouping of codes for similarities and difference, leading to the generation of descriptive themes, under which initial codes were consolidated to encapsulate their meaning; Producing final analytical themes derived from the descriptive themes, which tell the overall narrative in relation to the research aims.

Agreement for the final conceptual/analytical themes was reached through discussion and revision within the research team. NVivo (QSR International Pty Ltd., 2020) was utilised to facilitate synthesis and data storage.

## Results

### Study characteristics

The research aims/objective were identifiable for all 14 included studies. Table 5 shows the characteristics of the included articles. The UK was the predominant setting for the studies (*n* = 7), two studies were undertaken in Australia, four took place in the USA and one within Ireland. Thirteen of the studies used qualitative methodology, considered to meet the study objective. Marsack-Topolewsk and Samuel (2020) utilised mixed methodology appropriately to address their research aims. Eight of the studies used mixed populations comprising of varying participant combinations including care recipients, family carers, formal carers, and health care professionals (Carling-Jenkins et al., 2012; Furniss et al., 2012; Herron et al., 2020; Kerr & Wilkinson, 2006; Ryan et al., 2018; Sheth, 2019; Watchman, 2005; Wilkinson et al., 2005). Where studies utilised mixed populations the researcher (MH) was careful to extract the qualitative data attributable to the family and formal carers for synthesis.

**Table 5.** Study Characteristics of included articles.

Author	Date	Title	Country	Design	Population & Sample Size	Key Findings
Carling-Jenkins, Torr, Iacono, & Bigby	2012	Experiences of supporting people with Down syndrome and Alzheimer’s disease in aged care and family environments.	Australia	Semi structured interviews	*N* = not clearly defined.	• Changes in behaviour presentation and personality caused distress and confusion.
					Care recipient one’s carers= (manager of care facility, parents, behaviour support worker, team leader and staff from aged care facility.	• Overshadowing-behaviour changes attributed to down syndrome (DS) not AD.
					Care recipient two carer’s = psychiatry case manager, 3x care staff from aged care facility	• Overshadowing resulted in support and mismanagement of needs.
					Care recipient three = 2x siblings	• Family stressors included emotional/social, financial, and physical.
Coyle, Kramer & Mutchler	2014	Aging Together: Sibling Carers of Adults with Intellectual and Developmental Disabilities	USA	Semi structured	*N* = 15 adult siblings	• Increased caring demands.
				Interviews		• Lack of dementia knowledge
						• Carer sacrifices
						• Support and planning influences adjustment
Furniss, Loverseed, Lippold, & Dodd	2012	The views of people who care for adults with Down’s syndrome and dementia: a service evaluation.	UK	Semi structured interviews & Support service checklist	*N* = 13 (Sibling x2 Relatives non direct care x3, residential care staff x8)	• Knowledge differences formal carers and family carers.
						• Training, strategy and education needs
						• Dementia progression generates anxiety.
						• Family carers held a high responsibility and commitment to caring.
						• Emotional and physical stressors for family carers and relatives
						• Carer burden and resilience
Herron, Priest, & Read,	2020	Supporting people with an intellectual disability and dementia: A constructivist grounded theory study exploring care providers’ views and experiences in the UK.	UK	A constructivist grounded theory methodology utilising	*N* = 18 (Family carers x2, formal carers x8 and healthcare professionals x8. 5 participants interviewed twice)	• Overshadowing, lack of dementia knowledge can impact diagnosis.
				23 semi-structured interviews		• Person-centred approaches utilised.
						• Dementia approaches can conflict with Learning Disability approaches.
						• Different support structures for family and formal carers.
						• Training needs.
						• Strong attachments continuity of care valued.
						• Barriers to continuity of care
						• Carer burden, grief, and instability impact emotional and physical wellbeing.
Iacono, Bigby, Carling-Jenkins, & Torr	2014	Taking each day as it comes: staff experiences of supporting people with D own syndrome and Alzheimer’s disease in group homes.	Australia	Two semi structured face-to-face interviews conducted 6–12 months apart	*N* = 18 (Direct Care Staff and follow-up interviews after 6–9 months *N* = 15)	• Poor knowledge of dementia
						• Overshadowing
						• Limited, reactive and ad hoc strategies and support
						• Continuity of care
						• Lacking confidence
						• Barriers accessing external support.
Kerr & Wilkinson	2006	Responding to pain needs of people with a learning disability/intellectual disability and dementia: What are the key lessons?	UK	Informal Individual interviews	*N* = 86 residential and non-residential service provision.	• Overshadowing
					Support staff 49	• Dementia compounds communication difficulties.
					Psychiatrists 2	• Unhelpful beliefs about tolerance of pain.
					Psychologists 3	
					Managers 12	
					GPs 6	
					Community nurses 6	
					Occupational therapists 2	
					People with dementia 6)	
Marsack-Topolewski & Samuel	2020	Caregivers’ Perceptions of Family Quality of Life of Individuals with Developmental Disabilities Comorbid with Dementia: A Pilot Study	USA	Mixed methods. Online Quality of Life survey.	*N* = 13 Family caregivers	• External agencies support limited and barriers to access.
					Parent x10	• Psychological and social impact.
					Sibling x2	• Caregiver personal values.
					Nonrelative x1	
Marsack-Topolewski & Brady	2020	Experiences of Family Caregivers of Individuals with ID and Dementia	USA	Individual, semi-structured interviews	*N* = 6 (Family caregivers 1x sibling, 5x parents)	• Diagnosis challenges, overshadowing.
						• Difficultly identifying/accessing/utilising services for dual condition
						• Reluctance to access external support.
						• Carer responsibility to advocate.
						• Emotional impact
						• Rewarding caregiving
McLaughlin, & Jones	2011	‘It’s all changed: ’carers’ experiences of caring for adults who have Down’s syndrome and dementia	UK	Unstructured, face to face interview format	*N* = 6 (4 = sibling carers, 2 residential placement carers)	• Increased carer dependency/demands.
						• Changing carer information needs
						• Strong emotions attached to dementia diagnosis.
						• Diagnosis supports understanding generates anxiety.
						• Increased carer needs, support, and carer sacrifices.
Perera, B. D., & Standen, P. J.	2014	Exploring coping strategies of carers looking after people with intellectual disabilities and dementia.	UK	Focus groups & face-to-face interviews	*N* = 9 carers (6 = formal- residential and nursing home carers & 3 family carers)	• Narratives utilised for understanding identify and roles.
					Focus group- Learning Disability Nurses *n* = undisclosed	• Flexible and adaptive caring styles/strategies.
						• History and Established relationship.
						• Previous caring experience increased strategies for self-care and direct support.
						• Variation between formal and family carers coping strategies and resources.
Ryan, C., MacHale, R., & Hickey, E.	2018	Forgetting familiar faces. Staff perceptions of dementia in people with intellectual disabilities	Ireland	Focus Groups x2	*N* = 18 (Frontline staff members from day and residential services)	• Carers recognised cognitive/behavioural/physical symptoms of dementia.
						• Overshadowing and support implications
						• Challenging to balance decline, duty of care and independence
						• Continuity of care
						• Ageing in place
						• Emotional impact of caring.
						• Limited resources.
						• Training, information and support needs
						• Service user peers impacted.
						• Emotional impact.
						• Transition of family carers challenging
Sheth, A.J.	2019	Intellectual disability and dementia: perspectives on environmental influences. Quality in Ageing and Older Adults	USA	5 standard focus groups	*N* = 16	• Routine and structure
					4 adults with intellectual disabilities and dementia participated in 2 nominal group technique sessions.	• Multiple carer roles
					6 family carers 2x parents, 4 siblings	• Burdensome- behaviours and environment.
					6 residential placement staff	• Consistency of care
						• Training, information, and support needs
						• Resource and organisational barriers
						• Recognised need for specialist medical professionals
						• Behaviour challenges and strategies
Watchman	2005	Practitioner-Raised Issues and End-of-Life Care for Adults with Down Syndrome and Dementia	UK	Semi structured Interviews	*N* = 10	• Overlapping conditions
					8x residential services staff	• Difficulty balancing learning disability approach and dementia need.
					2x worked in palliative care settings.	• Carer training needs.
						• Environmental challenges.
						• Poor transition of care.
						• Unaware of palliative care services
Wilkinson, H., Kerr, D., & Cunningham, C.	2005	Equipping staff to support people with an intellectual disability and dementia in care home settings	UK	Interviews, focus groups and field notes (observations)	Direct care staff, managers, home residents, and relatives	• Caring commitment
					*N* = not disclosed	• Carer burden
						• Organisational structures and strategies
						• Lack of knowledge, training, and skills.
						• Inadequate environments
						• Overshadowing, pain mismanagement

Nine of the studies explored the care experiences of carers for people with a learning disability and dementia more broadly whereas four studies looked more purposefully at DS and dementia (Furniss et al., 2012; Iacono et al., 2014; McLaughlin & Jones, 2011; Watchman, 2005).

The predominant method of data collection was semi structured interviews (*n* = 7) (Carling-Jenkins et al., 2012; Coyle et al., 2014; Furniss et al., 2012; Herron et al., 2020; Iacono et al., 2014; Marsack-Topolewski & Brady, 2020; Watchman, 2005). Most of the data collection methods were clearly described. However, 4 studies only offered partial descriptions lacking details to consider if replicable and/or systematic (Kerr & Wilkinson, 2006; McLaughlin & Jones, 2011; Watchman, 2005; Wilkinson et al., 2005).

### Themes

Four conceptual themes emerged following analysis: *Knowledge and skills, Accessing support, Repercussions of dementia for carers, Influences of continuity of caring role*. These themes and supporting subthemes are presented below in Table 6, along with the illustrative quotes.

**Table 6.** Organisational chart of themes that emerged following data analysis with supporting quotations.

Theme	*Quotations*
**Knowledge and skills** Training, education, learning and development.	“I didn’t understand much about dementia…in our heads it was just something that happened to old people, not younger people with learning disability and Down syndrome…I think we just managed”(Herron et al., 2020, p. 1412).
	“The college course, it just made me question everything I was doing with the person with dementia…“(Herron et al., 2020, p. 1413, p. 1413)
	“she did everything wrong’ by telling him what to do, using confrontation, and telling him ‘no’” (Iacono et al., 2014, p. 527, p. 527)
	“She [the trainer] really enlightened us about the symptoms and things and a lot of good practice that we didn’t know about and this is when everybody thought oh look at all the mistakes we made…” (Wilkinson et al., 2005, p. 395).
Employed carer strategies and approaches.	“Yeah, well, we’ll take her to the toilet, if she’s still screaming we’ll give her a drink, if she’s still screaming, we’ll take her for a walk” (Iacono et al., 2014, p. 527, p. 527)
	“I really have no idea, of anything, because we, at times, really, clutch at straws? And a lot of it we ride on the seat of our pants” (Iacono et al., 2014, p. 527, p. 527)
	“It was like falling off the end of a cliff. We spent all out time trying to catch-up, everything we put into place was a month too late” (Watchman, 2005, p. 158, p. 158)
	“God has been good to us and we trust He will continue to help us with our challenges.” (Marsack-Topolewsk & Samuel, 2020, p. 64, p. 64)
**Accessing support** Overshadowing	“it’s hard to know whether it’s just a problem with their [intellectual] disability. I think we had a few years where we were very unsure.” (Herron et al., 2020, p. 1411, p. 1411)
	“They think it’s a behavioral issue when it’s really. . . the [dementia] is making them do it.” (Coyle et al., 2014, p. 309, p. 309)
	“him up to his old ways”(Kerr & Wilkinson, 2006, p. 71, p. 71)
	“we kept getting told by the doctor its part of her dementia. My argument is this, its part of her dementia but, let’s do something about it” (Iacono et al., 2014, p. 529, p. 529)
	“I think we go down the behaviour route before we go down the dementia route” (Ryan et al., 2018)
	“with ID and no spoken language, just not knowing if he’s upset, we don’t know why… you know, it’s even more of the guessing game… than it was prior to the dementia setting in.” (Marsack-Topolewski & Brady, 2020, p. 59)
Services and professional input	“The social worker has been very helpful he’s been good. He’s got respite for us you know every month we get four nights. It makes a big difference” (McLaughlin & Jones, 2011, p. 61).
	“I’ve no idea mate. I think [disability clinic] is the only one I know at the moment”(Iacono et al., 2014, p. 529, p. 529)
	“We believe that we might not pursue any extra care because my sister does not want to be in the hospital again […] the knowledge that they’ve given us and the resources to start planning for some that.” (Sheth, 2019, p. 186, p. 186)
	“It was frustrating because we knew there was things wrong. We knew that he was vulnerable at home, but we couldn’t get any of the social workers until things got really bad” (Herron et al., 2020, p. 1412).
	“I mean, I’m finding that I have to – that is, if they’re open to listening to me— I’m having to educate Tim’s [son] medical providers.“(Marsack-Topolewski & Brady, 2020, p. 58, p. 58)
	“actually finding some place that you have the confidence in” (Marsack-Topolewski & Brady, 2020, p. 58, p. 58)
	“This time round, there is a clear diagnosis….it feels like there’s a lot of support there”. (Furniss et al., 2012, p. 324, p. 324)
**Repercussions of the caring role** Increased caring demands	“That’s why I left my job. My school day was interrupted with phone calls about medical issues. I was going. . . every two weeks probably and. . . would. . . stay for three, or four or five days”. (Coyle et al., 2014, p. 306, p. 306)
	“Just there’s more. . . things to deal with. Before it was kind of like automatic pilot.“(Coyle et al., 2014)
	“she’d only use a fork now…everything has to be mashed together” … “they don’t drink as much, get dehydrated” (Ryan et al., 2018, p. 157).
	…everything changes with her now…care plans, risk assessments, all that’s been changed. Support levels have been changed with her, it’s a whole new routine with her depending on what day she’s having…. (Herron et al., 2020, p. 1413, p. 1413)
	“By the time you’ve been up the sixth time you’re getting cross, because you’re so tired”(Furniss et al., 2012, p. 323, p. 323)
Psychosocial well-being and quality of life	“That’s why I left my job. My school day was interrupted with phone calls about medical issues. I was going. . . every two weeks probably and. . . would. . . stay for three, or four or five days”. (Coyle et al., 2014, p. 306, p. 306)
	“You’re losing people…they’re just totally different” (Ryan et al., 2018, p. 157, p. 157)
	‘Yeah it does put a strain on you. No, we don’t have enough time for ourselves’.(Furniss et al., 2012, p. 323, p. 323)
	“Staff have found it a little bit hard emotionally because each week we see a little bit less of that person because the dementia’s changing him”. (Herron et al., 2020, p. 1413, p. 1413)
	They’re abusive to you…I think it’s a very difficult thing to cope with” (Furniss et al., 2012, p. 323, p. 323)
	“... I think that emotional involvement is the most difficult.“(Marsack-Topolewski & Brady, 2020, p. 59, p. 59)
	“…we are always in transit. Our lives get better, then worse... as my brother’s condition varies and/or the supports we receive change.” (Marsack-Topolewsk & Samuel, 2020, p. 64, p. 64)
**Influences of continuity in the caring role** Ageing in place	‘I’m told that it’s going to get worse and when that happens like I said as much as I love him he’ll have to go into care. I couldn’t do it no more it’s stressful for me and hurtful for me too (McLaughlin & Jones, 2011, p. 61, p. 61)
	“As long as we can do this financially and her health [is good], we’ll [support her].“(Coyle et al., 2014, p. 308, p. 308)
	“I still think it’s in his best interest and really it’s his right to actually have his own home too, to die in, or age in”(Iacono et al., 2014, p. 528, p. 528)
Environment and economic challenges	“The group home that he went to was not prepared for [someone with] Alzheimer’s disease. . . The transition wasn’t smooth. . . Things that we agreed would happen just didn’t happen.“(Coyle et al., 2014, p. 309, p. 309)
	” …she can no longer use the stairs and that’s awful. And she literally then had to leave her own house on that day…“(Ryan et al., 2018, p. 159).
	It’s actually what surrounds you when you are caring for somebody. The caring is fine. We have always cared and we hope to do the same for A [foster son] like the other two we cared for till they died. It is other structures that are not in place as they should be (Perera & Standen, 2014, p. 297).

#### Theme 1: Knowledge and skills

This theme encompasses the need to upskill and educate carers whilst recognising their current position of utilising their baseline skills and knowledge to manage the instability a dementia diagnosis creates.

##### Training, education, learning and development

The need to enhance the knowledge and skills of carers was referenced in eleven studies. The primary learning need for carers was dementia; understanding the condition, types of dementia, its progression, how to support the changing needs of their care recipients throughout the dementia journey (Carling-Jenkins et al., 2012; Coyle et al., 2014; Furniss et al., 2012; Herron et al., 2020; McLaughlin & Jones, 2011; Watchman, 2005; Wilkinson et al., 2005) and the link between the DS and developing dementia (Furniss et al., 2012; Herron et al., 2020; McLaughlin & Jones, 2011; Watchman, 2005).

Both family and formal carers recognised the need for tailored dementia training for the learning disability population (Iacono et al., 2014; Marsack-Topolewski & Brady, 2020). Further educational needs were identified regarding dementia and physical health, pain detection and management (Furniss et al., 2012; Kerr & Wilkinson, 2006; Wilkinson et al., 2005). Discrepancies regarding level of dementia knowledge between formal and family carers were noted, with family carers being at a lower level of awareness and understanding compared to formal carers (Furniss et al., 2012; McLaughlin & Jones, 2011). There were also differences within formal carers in relation to their acquired level of dementia understanding and experience and training undertaken, varying from college course to having no training at all (Furniss et al., 2012; Herron et al., 2020; Iacono et al., 2014; Wilkinson et al., 2005). The practice implications of insufficient carer education and training included the use of inappropriate strategies and approaches escalating behaviours, combined with a false confidence in their utilisation (Iacono et al., 2014; Wilkinson et al., 2005), increased emotional burden (Herron et al., 2020), and the employment of carers lacking the ability to meet the needs of someone with a dual diagnosis (Iacono et al., 2014; Marsack-Topolewski & Brady, 2020; Wilkinson et al., 2005), which can lead to high staff turnover (Watchman, 2005). Moreover carer’s lack of awareness of significance of changes in behaviour and functioning in relation to the onset of dementia impacted on care recipients receiving a timely diagnosis (McLaughlin & Jones, 2011).

The perceived benefits of dual diagnosis education and training included enhanced carer understanding and caring abilities (Carling-Jenkins et al., 2012; Furniss et al., 2012; Herron et al., 2020), sharing of knowledge and expertise between carers of how best to support their care recipients (Iacono et al., 2014; Perera & Standen, 2014) accessing external healthcare support (McLaughlin & Jones, 2011) and creating change in current practice through implementing learnt knowledge and strategies appropriate for dementia care (Herron et al., 2020; Wilkinson et al., 2005).

##### Employed carer strategies and approaches

Previous personal experience of caring for a family member with dementia was utilised to inform current care and practice (Furniss et al., 2012; McLaughlin & Jones, 2011; Watchman, 2005; Wilkinson et al., 2005). Active carer strategies split into two categories; strategies to support and manage the care recipient (Herron et al., 2020; Iacono et al., 2014; Perera & Standen, 2014; Sheth, 2019; Watchman, 2005; Wilkinson et al., 2005) and carer strategies for self-care and preservation (Carling-Jenkins et al., 2012; Furniss et al., 2012; Herron et al., 2020; Iacono et al., 2014; McLaughlin & Jones, 2011; Perera & Standen, 2014; Ryan et al., 2018; Watchman, 2005).

Carers employed learning disability informed strategies to support and manage dementia needs and behaviours (Iacono et al., 2014), with a person-centred approach underpinning their care (Herron et al., 2020; Iacono et al., 2014; Perera & Standen, 2014; Sheth, 2019; Wilkinson et al., 2005) for example playing a clients preferred music (Perera & Standen, 2014) and promoting routine and structure (Sheth, 2019). Trial and error are utilised by carers to manage and adapt to the unpredictability of presenting needs and behaviours (Iacono et al., 2014; Perera & Standen, 2014; Sheth, 2019). Communicating and sharing of information among carers was an important approach recognised by both family and formal carers to facilitate quality care (Sheth, 2019; Wilkinson et al., 2005).

Formal carers had greater availability and implementation of self-care strategies compared to family carers such as supervision, physically and emotionally distancing themselves, setting realistic expectations and goals (Perera & Standen, 2014), working shorter and flexible shifts (Wilkinson et al., 2005), and peer support to normalise and validate thoughts and emotions (Herron et al., 2020; Perera & Standen, 2014; Ryan et al., 2018). Family carers utilised their faith for support and hope to navigate the challenges within their caring role (Marsack-Topolewsk & Samuel, 2020; Perera & Standen, 2014).

#### Theme 2: Accessing support

This theme reflects the significant challenges carers face with accessing support. The overlapping of the conditions poses a particular conundrum for carers and professional with regards to the subsequent care they provide.

##### Overshadowing

Ten papers referenced overshadowing and overlapping of conditions (Carling-Jenkins et al., 2012; Coyle et al., 2014; Furniss et al., 2012; Herron et al., 2020; Iacono et al., 2014; Kerr & Wilkinson, 2006; Marsack-Topolewsk & Samuel, 2020; Marsack-Topolewski & Brady, 2020; Ryan et al., 2018; Wilkinson et al., 2005). Care recipient’s behaviours pose a significant challenge for both family and formal carers in relation to whether they are attributable to learning disability, personality, dementia, physical or mental health condition.

Overshadowing extended beyond carers and into the professional field, with carers’ dementia concerns regarding behavioural changes being disregarded and/or attributed to their learning disability, with carers having to push for concerns to be taken seriously (Herron et al., 2020; Marsack-Topolewski & Brady, 2020), to ensure that health needs are met (Iacono et al., 2014).

##### Services and professional input

Mixed experiences of accessing and utilising services and professionals were reported in eleven studies. Positive experiences included faster diagnosis and access to tailored information compared to previous caring experiences, when support is available (Furniss et al., 2012) and access to medication to support dementia behaviours that challenge and other health conditions (Iacono et al., 2014). Family carers utilise external support to take respite and support daily living activities (McLaughlin & Jones, 2011; Perera & Standen, 2014), for emotional support (McLaughlin & Jones, 2011) and for home adaptations (Furniss et al., 2012).

Carers reported disparity in accessing professional and external services and the input they provide (Iacono et al., 2014; Marsack-Topolewski & Brady, 2020). Referral pathways into health services reportedly differed between formal carers (Ryan et al., 2018), with staff working in residential settings having more direct routes then day centre staff. Family carers reported difficulty navigating social care organisations and services due their varying and changing structures and eligibility criteria (Marsack-Topolewski & Brady, 2020). A significant barrier for both sets of carers accessing external support was a lack of awareness of the health and social care services and support available (Herron et al., 2020; Iacono et al., 2014; McLaughlin & Jones, 2011; Watchman, 2005) and knowing when the right time is to ask for help (Furniss et al., 2012) or requests for support not being answered by palliative care professionals (Iacono et al., 2014). Level of service input varied within family carers, with some reporting having to fight to have professionals listen to their concerns and implement the right support (Herron et al., 2020; Iacono et al., 2014). Others reported being overwhelmed by the number of professionals involved (Furniss et al., 2012). There was a reported lack of confidence in health professionals and social service provider’s ability to deliver the care needed for the dual conditions (Carling-Jenkins et al., 2012; Coyle et al., 2014; Furniss et al., 2012; Iacono et al., 2014; Marsack-Topolewski & Brady, 2020). Such varied findings indicate the need for a review and revision of health and social care services to provide consistent and equitable care for service users.

#### Theme 3: Repercussions of dementia for carers

This theme highlights the compounding impact a dementia diagnosis has on carers who care for people with a learning disability.

##### Increased caring demands

Twelve studies referenced carers experiencing increased caring demands as dementia emerges and progresses (Carling-Jenkins et al., 2012; Coyle et al., 2014; Furniss et al., 2012; Herron et al., 2020; Iacono et al., 2014; Marsack-Topolewsk & Samuel, 2020; Marsack-Topolewski & Brady, 2020; Perera & Standen, 2014; Ryan et al., 2018; Sheth, 2019; Watchman, 2005; Wilkinson et al., 2005). Care recipient behavioural changes and reduced independence due to dementia made the caring role more challenging (Carling-Jenkins et al., 2012; Herron et al., 2020; Marsack-Topolewski & Brady, 2020; McLaughlin & Jones, 2011; Ryan et al., 2018). Risk management increased due to care recipients poor insight into their dementia related reduced abilities (Ryan et al., 2018).

Similar role changes were found between formal and family carers such as increased decision making and planning (Carling-Jenkins et al., 2012; Herron et al., 2020; Perera & Standen, 2014). Disorientation and night-time waking by care recipients increased daily caring/supervision hours and responsibilities for both sets of carers (Furniss et al., 2012; Iacono et al., 2014; Ryan et al., 2018; Wilkinson et al., 2005).

Family carers found themselves within an advocacy/researcher role for their care recipient as they tried to access support and/or resources for their care recipient (Marsack-Topolewski & Brady, 2020) and plan for their future (Carling-Jenkins et al., 2012). Family carers experienced increased caring demands though compound caring (Marsack-Topolewsk & Samuel, 2020), especially when parental carers can no longer provide support and require support themselves (Carling-Jenkins et al., 2012).

Formal carers based within day centres and residential settings faced increased demands balancing the additional needs of the care recipients with the established and subsequent needs of others within their care (Herron et al., 2020; Ryan et al., 2018), as well as balancing their duty of care, staff regulations and policies and care recipients independence and rights (Ryan et al., 2018; Sheth, 2019; Watchman, 2005).

##### Psychosocial well-being and quality of life

Thirteen studies made reference to the impact the development of dementia had on carers’ wellbeing and quality of life (Carling-Jenkins et al., 2012; Coyle et al., 2014; Furniss et al., 2012; Herron et al., 2020; Iacono et al., 2014; Marsack-Topolewsk & Samuel, 2020; Marsack-Topolewski & Brady, 2020; McLaughlin & Jones, 2011; Perera & Standen, 2014; Ryan et al., 2018; Sheth, 2019; Watchman, 2005; Wilkinson et al., 2005). Observing the decline in functioning and managing associated behavioural changes in care recipients had an emotional and psychological toll on carers (Carling-Jenkins et al., 2012; Coyle et al., 2014; Furniss et al., 2012; Herron et al., 2020; Iacono et al., 2014; Marsack-Topolewski & Brady, 2020; McLaughlin & Jones, 2011; Ryan et al., 2018; Wilkinson et al., 2005). Carers experienced feeling overburdened and burnout, (Furniss et al., 2012; Perera & Standen, 2014; Wilkinson et al., 2005). Some carers experienced and conceived these functional and behavioural changes due to dementia as a loss of the person (Furniss et al., 2012; Herron et al., 2020; McLaughlin & Jones, 2011; Ryan et al., 2018).

The emotion of guilt emerged in relation to carers not being able to meet dementia care needs (Herron et al., 2020; McLaughlin & Jones, 2011; Watchman, 2005; Wilkinson et al., 2005); their emotional response to care recipients and dementia behaviours that challenge (Furniss et al., 2012); parental caregivers passing on the caring role to siblings (Ryan et al., 2018); siblings being unable to fulfil promised caring commitment (Carling-Jenkins et al., 2012) and for dementia developing during their caring period (McLaughlin & Jones, 2011) and not during their parents care.

For family carers, there were significant social costs evidenced in relation to reduced independence (McLaughlin & Jones, 2011) and personal time (Furniss et al., 2012; McLaughlin & Jones, 2011), reduced social opportunities, leaving paid employment to assume full time caring role (Carling-Jenkins et al., 2012; Coyle et al., 2014; McLaughlin & Jones, 2011) and increased social isolation (Furniss et al., 2012; Marsack-Topolewsk & Samuel, 2020; McLaughlin & Jones, 2011).

Despite these recognised challenges and repercussions of the caring role, seven studies reported carers had a strong commitment to caring for individuals with a dual diagnosis (Furniss et al., 2012; Iacono et al., 2014; McLaughlin & Jones, 2011; Ryan et al., 2018; Wilkinson et al., 2005). This commitment was demonstrated by residential staff going beyond their role working extra hours and undertaking unpaid work (Furniss et al., 2012; Wilkinson et al., 2005), advocating for their care recipients rights to remain at home and receive appropriate care (Iacono et al., 2014; Marsack-Topolewski & Brady, 2020). Their commitment was underpinned by formal and family carers strong attachments to their care recipients (Herron et al., 2020; Iacono et al., 2014; Marsack-Topolewski & Brady, 2020; Wilkinson et al., 2005)

#### Theme 4: Influences of continuity of caring role

Carers discussed key factors which facilitate and challenge them in providing continued care for care recipients.

##### Ageing in place

Carers’ preferences for care recipients to remain in their home following diagnosis dementia and across the trajectory of the condition was reported in eight studies (Carling-Jenkins et al., 2012; Furniss et al., 2012; Herron et al., 2020; Iacono et al., 2014; Perera & Standen, 2014; Ryan et al., 2018; Watchman, 2005; Wilkinson et al., 2005). There was recognition by carers that this was in the best interests of the care recipient (Herron et al., 2020; Iacono et al., 2014; Ryan et al., 2018). This preference was underpinned for some carers by negative experiences of care recipients moving on and receiving poor care (Iacono et al., 2014). Barriers to remining at home were recognised by formal and family carers as lack of skills and ability to cope with dementia decline and increasing needs (Carling-Jenkins et al., 2012; Furniss et al., 2012; Herron et al., 2020; Iacono et al., 2014; McLaughlin & Jones, 2011; Wilkinson et al., 2005).

##### Environment, resources, and economic challenges

The environmental and logistical needs and barriers to caring for someone with a dual diagnosis were discussed by carers in ten studies (Coyle et al., 2014; Furniss et al., 2012; Herron et al., 2020; Iacono et al., 2014; Perera & Standen, 2014; Ryan et al., 2018; Sheth, 2019; Watchman, 2005; Wilkinson et al., 2005). Factors such as physically disabling, unsafe, cognitively overloading and overstimulating environments were acknowledged by carers. Carers expressed concern for care recipient’s safety within the home due to changing complex needs as dementia progressed (Ryan et al., 2018).

Carers reported limited resources and limiting organisational structures and governance were affecting care provision and continuity of care. Inhibiting factors for formal carers included staffing levels and ratios (Furniss et al., 2012; Iacono et al., 2014; Ryan et al., 2018; Sheth, 2019; Wilkinson et al., 2005); additional daily living and administrative tasks, staff rules and policies (Sheth, 2019); lack of care recipient background information on transfer to new placement (Perera & Standen, 2014; Ryan et al., 2018); poor understanding of dual diagnosis needs at senior level within organizations (Iacono et al., 2014) and carers lacking in end of life support training. Sibling carers highlighted a lack of planning and preparation to meet care recipients dementia needs impacted on care recipient transition and continuity of care (Coyle et al., 2014).

Both family and formal carers recognised the financial implications of a dementia diagnosis and its impact on care recipients, carers and continuity of care (Carling-Jenkins et al., 2012; Herron et al., 2020; Marsack-Topolewski & Brady, 2020). Carers a reported lack of funding cuts and funding affected care continuity and them accessing support. (Carling-Jenkins et al., 2012; Herron et al., 2020; Marsack-Topolewski & Brady, 2020).

## Discussion

This qualitative systematic review offers a novel and comprehensive exploration into carers’ experiences of caring for people with a learning disability and dementia. Previous systematic reviews synthesised solely professionals experiences (Cleary & Doody, 2017) and reviewed caregiving interventions utilised by carers to support care recipients with the dual diagnosis (Courtenay et al., 2010). This systematic review, however, highlights the challenges and consequences formal and family carers can experience when trying to deliver quality care for those with a dual diagnosis, and navigate the shifting demands of dementia. It also highlights key factors that support carers within their caring role. Acton et al.’s (2023) similar systematic review utilises only eight out of the fourteen studies included in this review, to map the challenges experienced by carers. Acton et al.’s (2023) identified domains, such as gaps in knowledge and skills, burden and increased care demands are evidenced with the emerging themes of this review. Using thematic synthesis, the present review provides a holistic exploration of carers’ experiences and facilitates a greater understanding and differentiation of formal and family carers experiences and needs.

The increased caring demands associated with dementia were strongly reported by both family and formal carers (Coyle et al., 2014; Herron et al., 2020; Marsack-Topolewsk & Samuel, 2020; McLaughlin & Jones, 2011). Research indicates time spent caring for a person with a learning disability significantly increases following the onset of dementia (Janicki et al., 2005; McCarron et al., 2002). McCarron et al. (2002) report time spent caring does not vary between mid and end stage dementia, but that caring roles change with dementia progression. Sutcliffe et al. (2017) further highlight the change in intensity of the caring role and the emotional impact due to dementia progression. They found carer burden was significantly associated with neuropsychiatric symptoms, which increases dependency on carers for support with daily living skills. Variation of caring role is reflected in our subtheme increased caring demands*,* with varying responsibilities referenced, such as increased planning and decision making (Perera & Standen, 2014) and increased supervision due to disorientation and night-time waking (Furniss et al., 2012; Iacono et al., 2014). Evidence indicates the caring role for individuals with a learning disability alone can be challenging (Antonsson et al., 2008; Tyrer et al., 2006), therefore greater research is needed to understand how learning disability carers psychologically adjust to the additional demands and the psychological impact of a dementia diagnosis. To our knowledge there is no predominant theoretical model of psychological adjustment following a dementia diagnosis (Brooker et al., 2017). With the added complexities of a learning disability, it would be helpful to have a deeper understanding of the facilitators and/or obstacles to adjustment for this niche carer population. In doing so, support and interventions could be tailored to promote early adjustment to reduce distress and support quality of life for carers and ultimately care recipients (Brooker et al., 2017; Sutcliffe et al., 2017).

Evidence showed carers needed to be upskilled and increase their knowledge of dementia, specifically in relation to its impact and interaction with learning disability (Herron et al., 2020; Iacono et al., 2014; Ryan et al., 2018; Sheth, 2019). Different educational and training needs were identified between family and formal carers, and between groups of formal carers. Some formal carers reportedly had a higher level of understanding and awareness in comparison to family carers (Furniss et al., 2012; Herron et al., 2020; McLaughlin & Jones, 2011). Differences between formal carers related to access to training, the quality of training provided and level of carer experience and understanding (Furniss et al., 2012; Wilkinson et al., 2005). Current evidence argues that formal dementia carers such as homecare workers need greater education and training to support the symptoms of dementia (Kamalraj et al., 2021; Polacsek et al., 2020). It could be argued that formally working in the field of learning disability may facilitate carers access to training. However, research exploring other significant comorbidities within the learning disability population similarly found limited or no training opportunities and significant education and training needs (Bates et al., 2004). Dementia specific training packages are available for learning disability formal carers with noted positive outcomes such as increased understanding, awareness of support, carer wellbeing and confidence working with dementia (Dicks et al., 2015; Fahey-McCarthy et al., 2009; Kalsy et al., 2007). However, findings from this systematic review suggest that the benchmark for formal carers’ skills and knowledge of dementia continues to be substandard, and for family carers even lower.

Given the identified differences in education and training, it is unspringing that variations were found in the strategies utilised by both carer groups. Formal carers had greater access to strategies and organisational processes that supported their wellbeing (Herron et al., 2020; Perera & Standen, 2014; Wilkinson et al., 2005). This may offer explanation as to why some evidence suggests dementia care home staff experience low to moderate levels of burnout and stress (Costello et al., 2019; Pitfield et al., 2011). Costello et al. (2019) suggests specific groups of carers, (e.g., caring for individuals presenting with aggressive behaviour) may be more vulnerable to stress and burnout. This is pertinent for learning disability carers as well as dementia carers as “behaviours that challenge” are observed amongst the learning disability population and have been found to significantly impact on carer stress and wellbeing (Ryan et al., 2019). The *employed carer strategies* reported in this review, such as utilising learning disability approaches (Iacono et al., 2014), and self-care strategies (Perera & Standen, 2014), support and add to the evidence base as factors that can help shield against reduced carer psychological wellbeing (Cooper et al., 2016). However, despite employing strategies to manage care recipient changes and behaviours and promote self-care, both carer groups reported on the negative impact dementia had on their psychosocial wellbeing (Carling-Jenkins et al., 2012; McLaughlin & Jones, 2011).

Both sets of carers demonstrated a strong commitment to their caring role regardless of the identified challenges and difficulties. This was further demonstrated through their preference for care recipients to remain in their own home (Herron et al., 2020; Iacono et al., 2014; Ryan et al., 2018). However, environmental, resource and economic factors were significant barriers to being able to provide the desired continuity of care. These findings are consistent with barriers identified by carers for people with dementia (without a learning disability) (Ball et al., 2004; Giebel et al., 2021; Hoof et al., 2010; Thoma-Lürken et al., 2018). This review highlights that carers have mixed experiences of services and professional input due to difficulty navigating pathways, and disparity in provision and available services. The overshadowing of conditions was recognised and linked to diagnosis difficulties and insufficient professional support. In addition the benefit of specialised services, compared to generic services, meeting the needs of people with a learning disability is evidenced (Jess et al., 2008). From a professional perspective, dementia pathways set up in community learning disability teams which encompasses screening and intervention, indicate effective multidisciplinary working to support the needs of people with a learning disability and their carers (Chapman et al., 2018). However, our findings based on the carer’s perspective would challenge this, highlighting the need for greater parity across services and further research exploring the needs of this carer population.

A domino effect can be observed in the emerged themes of this review. The cumulative effect of a lack of wider support from health and social care services, and the absence of carer knowledge, skills and resources to address the development of dementia significantly impacts on carer experience. The relationship between carers and care recipients with a learning disability is reciprocal and interdependence is evident (Fulton et al., 2021; Gove et al., 2017; Williams & Robinson, 2001). Given the findings of this review, future research should look to explore how people with a dual diagnosis experience their carer relationships and the quality of care received following the development of a dementia diagnosis. This will complement the carer reports evidenced in research synthesised in this review, and may enable professionals and services to ascertain what additional support may be needed for the carer dyad to continue to facilitate independence and personhood for individuals with a learning disability and dementia.

## Limitations

The synthesis utilised for this review could be considered a limitation, as themes have been developed from coding quotes without the context from which they originated. Inclusion criteria were set to only include studies written in English. The studies included are only representative of Western countries and practice. There is increasing ethnic diversity in western countries (Schneider & Heath, 2020; World Population Review, 2023) and so future research should seek to incorporate and identify ethnic similarities and differences of carers experiences. Moreover, future reviews should seek to synthesise the findings of low- and middle-income countries to identify cultural differences that might influence carer experiences and inform practice.

## Conclusions

There are significant recognised similarities and differences between family and formal carers as they strive to support people with a dual diagnosis to lead full and flourishing lives. Training and educational needs of all carers should be addressed to support the wellbeing of both carers and their care recipients to enhance quality of care. Training must be tailored to meet the varying educational levels of both carer groups, and their psychological readiness to receive it, with training sufficiently evaluated to inform how it has impacted carers and care recipient experiences of care. Disparity between family and formal carer strategies were reported, with formal carers perceivably in an advantageous position to access strategies and organisational processes that support their role and wellbeing. Changes in caring role demands were reported by both carer groups as dementia progresses, with care recipient dependency, assessment, planning, and supervision increasing. Despite reported challenges of their caring role, both carers demonstrated a desire for continuity of care and for carer recipients to remain in their own homes. Additional research, and professional and service consideration, is needed to address environmental and economic barriers to facilitate ageing in place. Greater parity across services is needed regarding, pathways, accessibility, and professional input. This, combined with carers adequately educated and trained to support carer recipients with a dual diagnosis, may help to facilitate the timely diagnosis of dementia required. Further research exploring the experiences of people with dementia and a LD about their care needs and carer relationships is needed to shape and direct services, training, and delivery of care.
